# The efficacy and safety of Epimedium in the treatment of primary osteoporosis: a systematic review and meta-analysis

**DOI:** 10.3389/fmed.2025.1675160

**Published:** 2025-09-19

**Authors:** Xian-Quan Zhang, Zhen-Hui Hua, Chu-Jia Huang, Hai-Yun Chen, Zong-Ren Hu, Mei-Ren Zhang, Jun Liu

**Affiliations:** ^1^The Second Clinical College of Guangzhou University of Chinese Medicine, Guangzhou, China; ^2^Bone and Joint Research Team of Degeneration and Injury, Guangdong Provincial Academy of Chinese Medical Sciences, Guangzhou, China; ^3^School of Traditional Chinese Medicine, Hunan University of Medicine, Huaihua, China

**Keywords:** Epimedium, primary osteoporosis, meta-analysis, bone mineral density, plant natural products

## Abstract

**Background:**

Primary Osteoporosis (POP) is a global public health issue, and traditional medications have long-term safety concerns. Epimedium, a kidney-tonifying traditional Chinese medicine (TCM), has the potential to regulate bone metabolism through multiple targets, but clinical evidence is scattered and inconclusive.

**Objective:**

This systematic review and meta-analysis aim to evaluate the efficacy and safety of Epimedium and its active components in treating POP.

**Method:**

Computer searches were conducted in multiple domestic and international databases, including PubMed, Embase, Cochrane Library, China National Knowledge Infrastructure (CNKI), and Wanfang Database, to collect randomized controlled trials (RCTs) comparing the use of Epimedium as an adjunct or alternative therapy with traditional drug treatments for POP. Literature was screened according to predefined inclusion and exclusion criteria, quality assessments were performed on eligible articles, relevant data were extracted, and statistical analysis was conducted using RevMan 5.4 software.

**Results:**

A total of 10 RCTs were included, with a final inclusion of 890 cases, 448 in the experimental group and 442 in the control group. Meta-analysis suggests that the overall efficacy rate of the Epimedium group was significantly higher than that of the control group (OR = 3.80; 95% CI: 2.27,6.37; *p* = 0.0001). Compared with the control group, Epimedium group’s Lumbar vertebra bone mineral density (BMD) (SMD = 1.15; 95% CI: 0.61,1.70; *p* < 0.0001), Femoral neck BMD (SMD = 1.11; 95% CI: 0.58,1.65; *p* < 0.0001), Distal radius BMD (SMD = 1.27; 95% CI: 0.57,1.98; *p* = 0.0004), and Metacarpal BMD (MD = 0.04; 95% CI: 0.04,0.12; *p* < 0.0001) all showed significant improvement, with a shorter time to relief of lower back pain (MD = -11.38; 95% CI: −12.63, −10.12; *p* < 0.00001). Serum alkaline phosphatase (ALP) was significantly reduced (MD = -8.78; 95% CI: −12.80, −4.77; <0.0001), while bone-specific alkaline phosphatase (BALP) increased (MD = 6.73; 95% CI: 3.32,10.14; *p* = 0.0001). Adverse reactions were low, mainly mild gastrointestinal reactions or skin allergies.

**Conclusion:**

Epimedium can effectively improve bone density and clinical symptoms in patients with POP, with good safety, making it a potential alternative or adjunctive treatment option, but more high-quality studies are needed to verify long-term efficacy.

## 1 Introduction

Osteoporosis (OP) is a systemic bone disease characterized by reduced bone mass and destruction of bone microstructure. The core pathophysiological mechanism is the disruption of the dynamic balance between bone resorption and bone formation, leading to increased bone fragility and significantly higher fracture risk. Based on the pathogenesis and population characteristics, the two most common types of primary osteoporosis (POP) include postmenopausal OP and senile OP. The former is mainly caused by a sharp drop in estrogen levels, which triggers hyperactivity of osteoclasts, while the latter is closely related to age-related decline in osteoblast function and abnormal vitamin D metabolism (1). According to epidemiological studies (2), the proportion of female patients is much higher than that of males, with varying incidence rates across different regions in China, but overall it remains at a high level, and the incidence continues to increase with age. The 2017 IOF European Audit Report (3) showed that approximately 200 million people worldwide suffer from OP, with an incidence rate as high as 30% among women over 50 years old and about 20% among men. OP causes more than 8.9 million fractures globally each year, with over one-third of these fractures occurring in Europe, creating significant treatment gaps and imposing substantial social and economic costs. In China, according to an epidemiological study on the incidence of OP in Beijing over the past decade (4), the incidence of OP among men ranges from 12.42 to 19.38%, while for women it is between 30.48 and 36.47%, indicating a higher incidence than in men. Over the past decade, both men and women have had relatively high rates of low bone mass, with men at 62.92 to 76.54% and women at 77.04 to 82.98%. The overall population incidence rate is between 72.61 and 81.22%. Additionally, studies have shown that although women are more prone to low bone density, the consequences of OP in men are more severe. Men have a higher mortality and morbidity rate for hip fractures compared to women, and their disease burden is even slightly greater than that of women (5). With population aging and increased life expectancy, the prevalence of OP has significantly risen. It is expected that the disease burden of OP and osteoporotic fractures will continue to increase, becoming a health issue affecting the global elderly population.

Currently, the conventional treatments for OP include bisphosphonates, selective estrogen receptor modulators (SERMs), RANKL inhibitors such as denosumab, and parathyroid hormone analogs like teriparatide (6). Although these drugs can effectively inhibit bone resorption or promote bone formation, their long-term use has significant limitations (7). For example, the incidence of flu-like symptoms such as joint pain, headache, and arthralgia caused by bisphosphonates is as high as 54.30%, and they may also induce atypical femoral fractures and jaw necrosis (8). Hormone replacement therapy (HRT) increases the risk of breast cancer and cardiovascular events (9). The high cost of biologics limits their widespread application. Currently, there is no complete cure for OP, and long-term use of medications can lead to severe adverse reactions. Therefore, exploring alternative therapies that combine efficacy with safety, developing new drugs, especially those based on natural products, to effectively treat OP while minimizing adverse reactions, has become a research hotspot in recent years.

In traditional Chinese medical texts, although the term “OP” is not explicitly mentioned, based on clinical manifestations, it can be categorized under conditions such as “bone atrophy,” “bone dryness,” and “bone arthralgia.” According to TCM theory, “the kidneys govern bones,” and kidney essence deficiency is the core pathogenesis of POP. Epimedium, a plant belonging to the genus Epimedium in the family Berberidaceae, has been regarded by TCM practitioners since ancient times as an essential herb for “tonifying the kidneys and strengthening bones.” The *Compendium of Materia Medica* records its benefits: “it nourishes essence and qi, strengthens tendons and bones, and tonifies the waist and knees.” Throughout history, physicians have widely used it to treat bone atrophy, soreness and weakness in the waist and knees, and other symptoms of kidney deficiency. Modern medicine also extensively uses decoctions, extracts, and formulations (such as Xianling Guobao capsules) of Epimedium for the clinical treatment of POP, achieving good clinical outcomes. Epimedium restores bone metabolic balance through “tonifying the kidneys and replenishing marrow,” aligning with the theoretical framework that “the kidneys govern bones.” Modern pharmacological studies have shown that the main active components of Epimedium, such as the flavonoid compounds Icariin (ICA), Epimedin (Epi), and Icaritin (ICAR), possess the potential to regulate bone metabolism through multi-target regulation. The mechanisms of action primarily include: ① Promoting osteogenesis differentiation: by activating BMP-2/RUNX2 and PI3K/AKT signaling pathways, the expression of Runx2, OCN, PI3K, AKT1 and other key osteogenic transcription factors and proteins was increased, and the differentiation of bone marrow mesenchymal stem cells to osteoblasts was stimulated (10); ② Inhibiting osteoclast activity: It downregulates the NF-κB and MAPK pathways, reducing RANKL-induced osteoclastogenesis, while also inhibiting the expression of bone resorption markers such as TRAP and CTSK (11); ③ Regulating the bone immune microenvironment: For example, it inhibits caspase-1 and signaling pathways mediated by NLRP3 inflammasomes, thereby reducing LPS-induced apoptosis levels and mitigating the negative impact of chronic inflammation on bone metabolism (12). Animal experiments further confirm that Icariin has estrogen-like effects, capable of inhibiting RANKL-induced osteoclast bone resorption function through the estrogen receptor ERα (13), significantly improving bone density and biomechanical properties in ovariectomized rats (14). Therefore, Epimedium has a long history of use in treating OP, and its role in POP treatment has been practically validated, demonstrating broad application potential.

However, the clinical translation of Epimedium remains challenging. There is a wealth of basic research on Epimedium’s treatment of POP, but fewer clinical trials, most of which are limited to small, single-center studies. Additionally, reports on efficacy evaluation metrics are inconsistent. Moreover, safety data for Epimedium glycosides are insufficient; some studies mention mild gastrointestinal reactions, but lack systematic assessments, and evidence-based medical literature is scarce. Therefore, we conducted a systematic review and meta-analysis of the effectiveness and safety of Epimedium in the treatment of POP, providing a reference for its rational use in clinical practice.

## 2 Materials and methods

This meta-analysis was conducted in strict accordance with the Preferred Reporting Itemsfor Systematic Reviewsand Meta Analyses (PRISMA) statement (15).

### 2.1 Search strategy

Two researchers independently searched multiple databases, including PubMed, Embase, Cochrane Library, China National Knowledge Infrastructure (CNKI) and Wanfang Database. The search period ranged from the database establishment to June 30, 2025. English search terms included “Epimedium,” “icariin,” “icaritin,” “epimedin,” “Osteoporosis,” “Osteoporosis, Postmenopausal” and “Senile Osteoporosis” and “Randomized controlled trial,” etc.; Chinese search terms included “淫羊藿,” “骨质疏松,” “临床研究.” A combination of subject terms and free text was used for searching, with appropriate adjustments made according to the characteristics of different databases. For example, the search queries in PubMed and CNKI are as follows: CNKI:
SU = (淫羊藿) and SU = (骨质疏松) and SU = (临床研究).
Pubmed:
#1:(((((Epimedium[Title/Abstract]) OR (Epimedium total flavonoid[Title/Abstract])) OR (Epimedium flavonoid[Title/Abstract])) OR (icariin[Title/Abstract])) OR (icaritin[Title/Abstract])) OR (epimedin[Title/Abstract]).
#2:((Osteoporosis[Title/Abstract]) OR (Osteoporosis[Title/Abstract] OR Postmenopausal[Title/Abstract])) OR (Senile Osteoporosis[Title/Abstract]).
#3: #1 and #2.
Filters: Randomized Controlled Trial.


### 2.2 Include inclusion and exclusion criteria

Inclusion criteria: ① Subjects: Clearly meet the POP diagnostic criteria (DXA bone density T-value≤ − 2.5); ②Interventions: Epimedium alone, extracts, or compound containing≥50% Epimedium, which can be combined with or not combined with other treatments; Control group: Any routine treatment; ③Study type: Must be a clinical randomized controlled trial; ④Literature must report outcomes related to clinical efficacy or safety evaluation.

Exclusion criteria: secondary OP, non-Chinese and English literature, repeated publication or incomplete data; review, experience summary, animal experiment, etc.

### 2.3 Outcome measures

Relevant outcome indicators: including overall efficacy, bone mineral density (BMD), which includes lumbar BMD, femoral neck BMD, distal radius BMD, and metaphyseal joint BMD; serum biochemical indicators include blood calcium (Ca^2+^), blood phosphorus (P), serum alkaline phosphatase (ALP), bone alkaline phosphatase (BALP), type I collagen cross-linked C-terminal peptide (CTX), and osteocalcin (BGP); time to relief of lower back pain and incidence of adverse reactions.

### 2.4 Literature screening and quality evaluation

Two researchers independently read the titles and abstracts of the retrieved literature, removing duplicates, and initially screened for potentially eligible articles based on inclusion and exclusion criteria. They then proceeded to read the full texts and determined the final list of included articles according to these criteria. Any disagreements during the screening process were resolved through discussion or consultation with a third researcher. The researchers standardized the data extraction from the included articles. Data extraction included basic research information, including the first author, year of publication, intervention, sample size, age, disease course, outcome measures, duration of treatment, outcome measures, and information related to literature quality assessment. The Cochrane bias risk assessment tool was used to evaluate the quality of the included articles, covering aspects such as random sequence generation, concealed allocation, blinding, completeness of data, selective reporting bias, and other biases. This evaluation tool assesses bias risks in five areas; if all five areas have low risk, the overall bias risk is low. If any one area has high risk, or if multiple areas have potential risks, the overall risk is high. Clinical RCTs that do not meet either of these two conditions may still have bias risks.

### 2.5 Data synthesis and statistical analysis

We used the RevMan 5.4 software for statistical analysis. For quantitative data, mean difference (MD) or standardized mean difference (SMD) and their 95% confidence intervals (CI) were used as measures of effect; for categorical data, odds ratio (OR) and its 95% CI were used as measures of effect. The *I*^2^ statistic was used to assess heterogeneity between studies. If *I*^2^ ≤ 50%, a fixed effects model was used for Meta-analysis; if *I*^2^ > 50%, the sources of heterogeneity were analyzed, and a random effects model was used when necessary.

### 2.6 Sensibility analysis and publication bias

We conducted sensitivity analyses to address potential heterogeneity and determine the robustness of the results. This was done by sequentially excluding each document, which increases the heterogeneity of each result to determine whether specific characteristics would alter the overall impact of each result. In this study, sensitivity analysis was performed in comparisons that included at least three articles. The funnel plot was used to determine whether there was publication bias in the included studies.

## 3 Results

### 3.1 Literature search results

**Figure 1:**
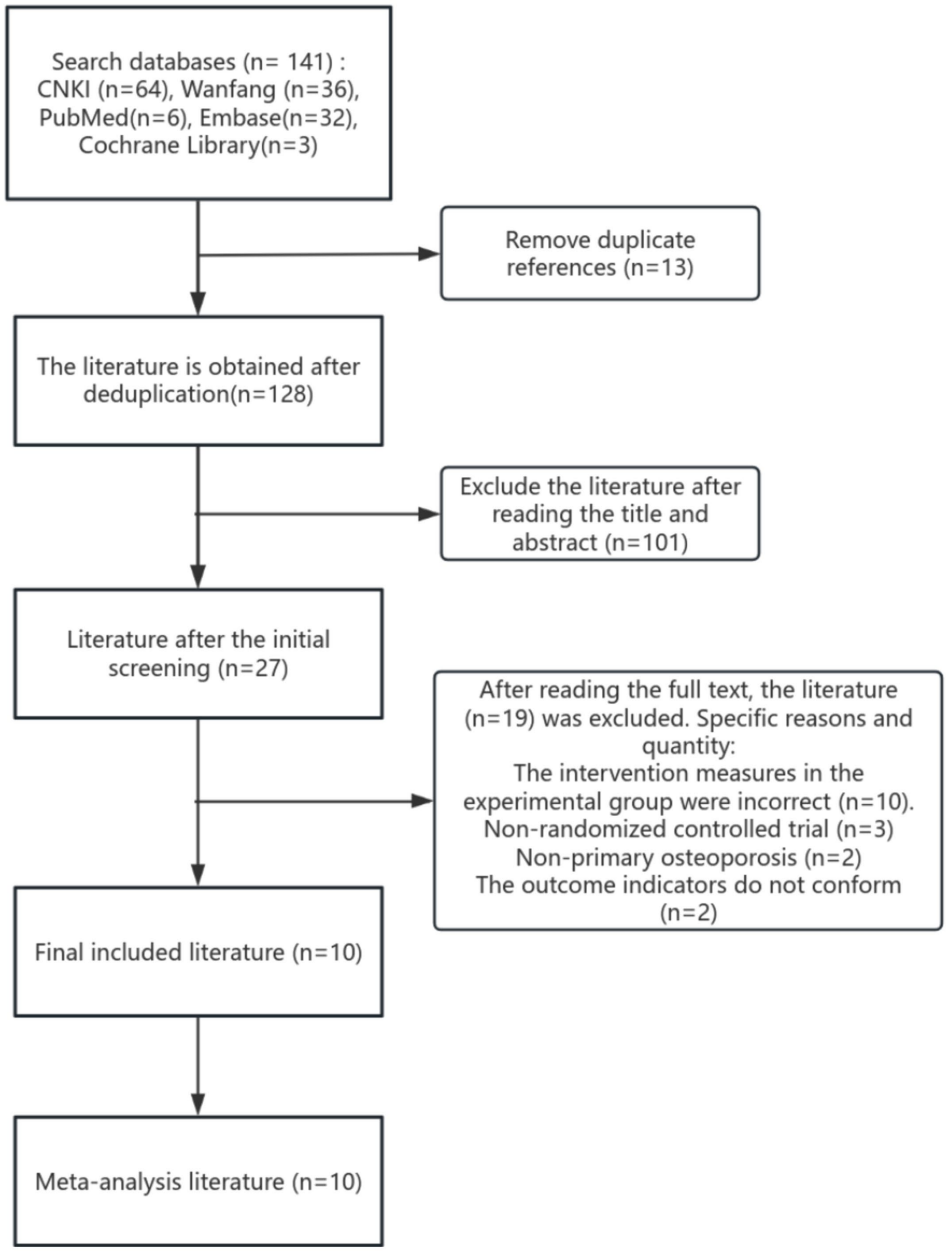
Flow chart for inclusion in the study.

A preliminary search yielded a total of 141 articles, including 64 from CNKI, 36 from Wanfang Database, 6 from PubMed Database, 32 from Embase Database, and 3 from Cochrane Library Database. After removing duplicates and reading the full texts, 10 articles were ultimately included (16–25), with 3 articles (16–18) being English articles and the remaining 7 articles(19–25), being Chinese articles. The literature search process and reasons for exclusion are shown in Figure 1. All 10 articles included in this study are clinical RCTs. A total of 905 cases were included, with 455 in the experimental group and 450 in the control group. One study (19) included 15 cases of dropouts due to loss to follow-up, with 7 in the experimental group and 8 in the control group. The other studies did not include any dropouts due to loss to follow-up. In total, 890 cases were included, with 448 in the experimental group and 442 in the control group. The basic characteristics of the included articles are listed in Table 1.

**Table 1:** Basic characteristics of the included literature.

Author	Year	Intervention		Sample size		Average age (years)		Duration (years)		Outcome	Follow-up (months)
		Experimental	Control	Experimental(M/F)	Control (M/F)	Experimental	Control	Experimental	Control		
Zhou (24)	2024	Epimedium total flavonoid capsules + alendronate sodium tablets	Aronophen sodium tablets	70 (36/34)	70 (35/35)	63.37 ± 43.89	63.18 ± 5.23	4.25 ± 1.41	4.19 ± 1.32	Total efficiency,lumbar BMD, femoral neck BMD, distal radius BMD, OC, PICP, BALP, CTX, TRACP, TNF-*α*, IL-17, GDF15	6
Liu (25)	2019	Extract of Epimedium	Calcium carbonate D3 tablets	61 (16/45)	61 (20/41)	67.46 ± 4.32	68.66 ± 6.21	17.67 ± 2.13	16.21 ± 1.98	Lumbar BMD, femoral neck BMD, metaphyseal joint BMD, ALP, Ca^2+^, P	3
Tu (21)	2018	Epimedium + Danxiankang bone capsules	Danxiankang bone Capsules	55 (21/34)	55 (23/32)	NA	NA	71.65 ± 4.01	70.38 ± 3.42	Lumbar BMD, femoral neck BMD, BGP, Ca^2+^, ALP, VAS	3
Tu (19)	2017	Compound Epimedium oral solution + alendronate sodium + Calcium D tablets	alendronate sodium + Calcium D tablets	58 (16/42)	58 (15/43)	61.8 ± 4.9	62.1 ± 5.5	1.6 ± 1.2	1.5 ± 1.2	Total efficiency, lumbar BMD, femoral neck BMD, metaphyseal joint BMD, BALP, PINP, S-CTX, ALP, BGP, OQOLS scale and TCM syndrome score	12
Zeng (23)	2017	Epimedium	Calcified triol soft capsules	45 (15/30)	45 (15/30)	74.5 ± 3.3	74.3 ± 3.2	NA	NA	Total efficiency, time to relieve lower back pain, lumbar BMD, femoral neck BMD, distal radius BMD, VAS	3
Zhu (22)	2014	Epimedium granules + Caltric D tablets	Caltric D tablets	40 (12/28)	40 (13/27)	NA	NA	NA	NA	Total efficiency,time to relieve lower back pain, lumbar BMD, femoral neck BMD, distal radius BMD	6
Zhao (20)	2003	Epimedium	Premarin see conjugated estrogen	15 (0/15)	10 (0/10)	NA	NA	NA	NA	Total efficiency,lumbar BMD, Ca^2+^, P, ALP	3, 6
Zhang (17)	2007	Eupolypharmic flavonoid compounds (EPFs) extracted from Epimedium	Placebo	50 (0/50)	50 (0/50)	64 ± 4	63 ± 3	NA	NA	Lumbar BMD, femoral neck BMD, DPD, OC, E2, Endometrial thickness	12, 24
Shou (16)	2009	Total flavonoids of Epimedium	Gushukang Capsules	32 (5/27)	32 (6/26)	62 ± 6	62 ± 7	NA	NA	Lumbar BMD, femoral neck BMD, wards triangle BMD, g reater trochanter BMD, left hip, Ca^2+^, P, ALP	6
Yong (18)	2021	Total flavonoids of Epimedium	Placebo	29 (0/29)	29 (0/29)	56.9 ± 11.8	57.0 ± 11.6	NA	NA	PINP, CTX, BALP, TRAF6	0.75, 1.5, 2

### 3.2 Quality evaluation of literature

**Figure 2:**
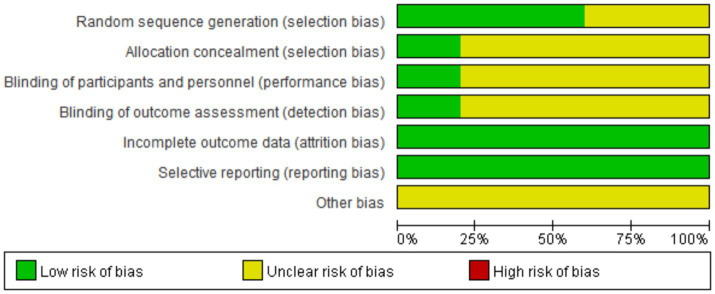
Evaluate the risk of bias in inclusion studies.

**Figure 3:**
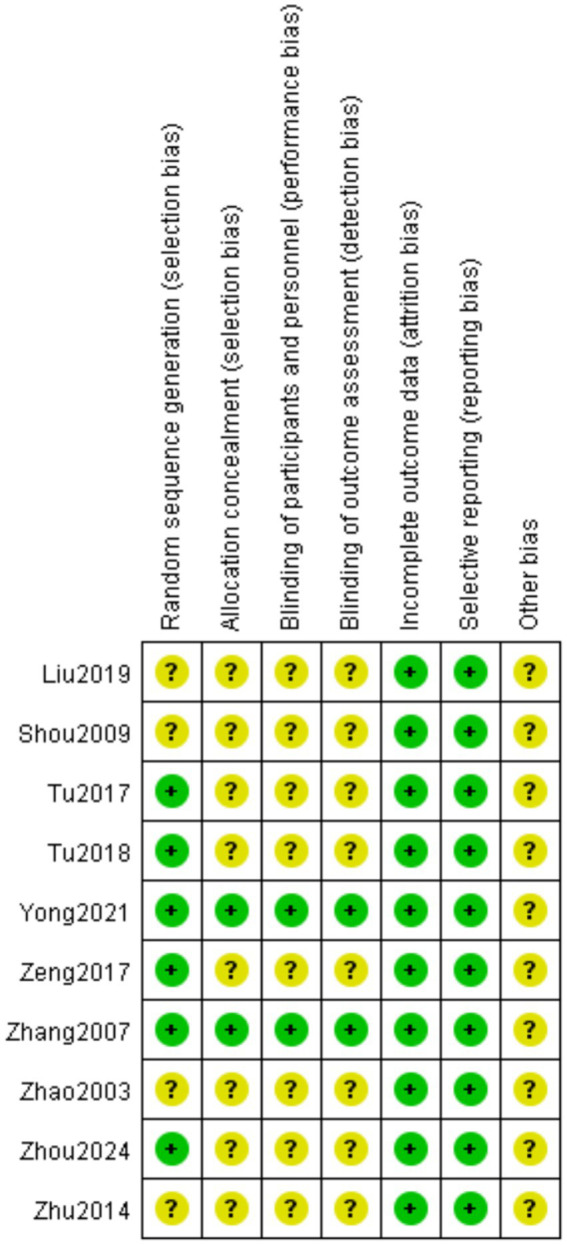
Evaluate the risk of bias in inclusion studies.

According to the Cochrane Randomized Controlled Trials (RCTs) Bias Risk Assessment Tool 2.0, the 10 included RCTs (16–25) were evaluated. The subjects of the literature studies had comparable baseline levels but exhibited varying degrees of bias. The risk of bias for the 10 included studies is shown in Figure 2. Two studies (17, 18) were rated as low risk overall. In terms of blinding implementation, three studies (20, 22, 25) only mentioned randomization without specifying practical allocation methods, classified as “unknown risk”; four studies (17, 19, 23, 24) used a random number table for grouping, one study (18) used a random envelope method for grouping, and one study (21) used a lottery system for grouping, all classified as “low risk.” Regarding allocation concealment, only two RCTs (17, 18) detailed the specific use of allocation concealment methods, classified as “low risk”; two studies only mentioned double-blind (17, 20), while the rest did not mention blinding, classified as “unknown risk.” In terms of data completeness, all 10 studies (16–25), reported according to the protocol and provided clear explanations for follow-up and dropouts, classified as “low risk.” In terms of selective reporting, none of the 10 studies (16–25) could be judged from the original text whether there were other sources of bias, classified as “unknown risk.” The results of the bias risk assessment are shown in Figures 2, 3.

### 3.3 Outcome

In the included studies, six studies (19–24) observed the overall effectiveness rate of clinical outcomes. Nine studies (16, 17, 19–25) evaluated BMD as one of the indicators of clinical results. Four studies (16, 20, 21, 25) reported Ca^2+^ levels, three studies (16, 20, 25) reported P levels. Five studies (16, 19–21, 25) reported ALP levels. Two studies (19, 24) reported BALP levels. Three studies (18, 19, 24) reported CTX levels. A total of two studies (19, 21) observed BGP. Three studies (20, 22, 23) observed the time it took for patients to achieve relief from lower back pain after treatment. Two studies (18, 24) mentioned adverse reactions that occurred during the study. Other results that were insufficient for meta-analysis were also described and recorded as part of the systematic review.

#### 3.3.1 Total effective rate

**Figure 4:**
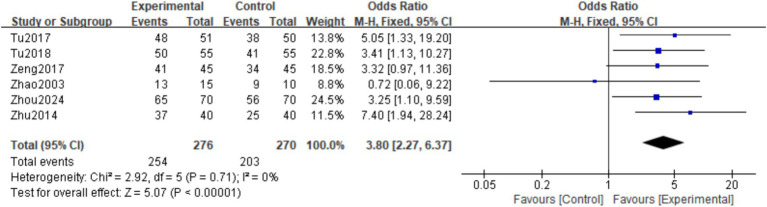
Forest plot of the total effective rate.

A total of six studies (19–24), observed the overall response rate at the final follow-up. The homogeneity among the studies was good (*I*^2^ = 0%, *p* = 0.71), so a fixed effects model was used. Meta-analysis showed that the overall response rate in the experimental group was significantly higher than in the control group. The difference was statistically significant (OR = 3.80; 95% CI: 2.27,6.37; *p* = 0.0001) (Figure 4).

#### 3.3.2 Bone mineral density

##### 3.3.2.1 Lumbar vertebra BMD

**Figure 5:**
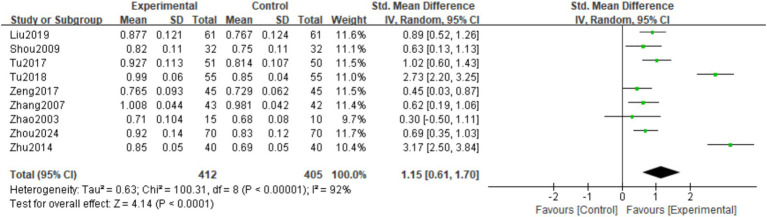
Forest plot of the lumbar vertebra BMD.

A total of 9 studies (16, 17, 19–25) observed Lumbar vertebra BMD at the final follow-up. There was significant heterogeneity among the studies (*I*^2^ = 92%, *p* < 0.00001), so a random effects model was used. The meta-analysis showed that the Lumbar vertebra BMD at the final follow-up in the experimental group was significantly higher than in the control group. The difference was statistically significant (SMD = 1.15; 95% CI: 0.61,1.70; *p* < 0.0001; Figure 5).

##### 3.3.2.2 Femoral neck BMD

**Figure 6:**
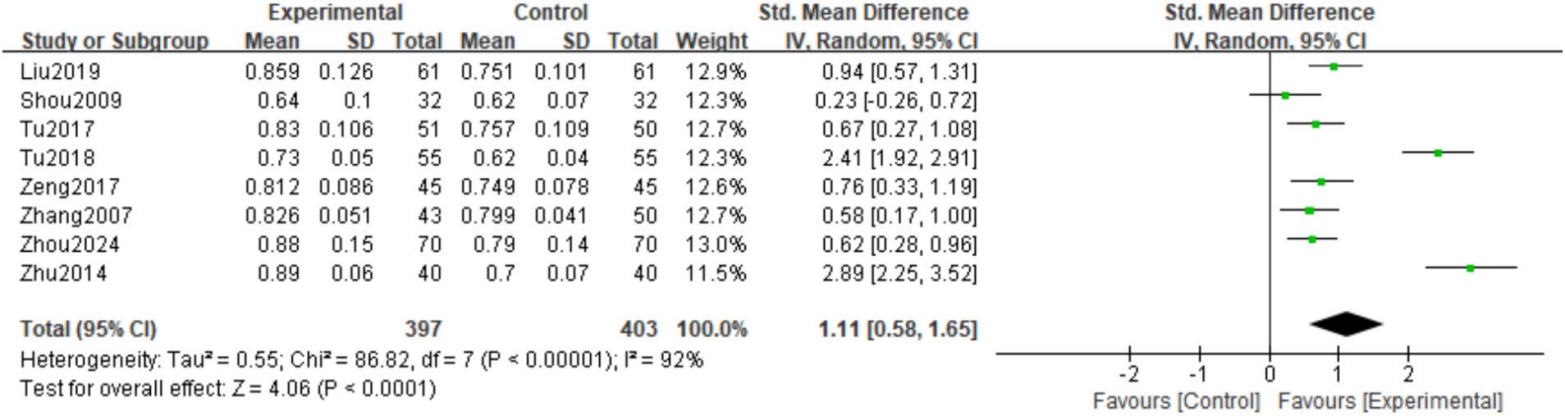
Forest plot of the femoral neck BMD.

A total of 8 studies (16, 17, 19, 21–25) observed Femoral neck BMD at the last follow-up. There was significant heterogeneity among the studies (*I*^2^ = 92%, *p* < 0.00001), so a random effects model was used. The meta-analysis showed that the Lumbar vertebra BMD at the last follow-up in the experimental group was significantly higher than in the control group. The difference was statistically significant (SMD = 1.11; 95% CI: 0.58,1.65; *p* < 0.0001; Figure 6).

##### 3.3.2.3 Distal radius BMD

**Figure 7:**
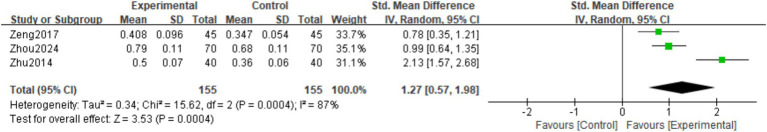
Forest plot of the distal radius BMD.

A total of three studies (22–24) observed the Distal radius BMD at the last follow-up. There was significant heterogeneity among the studies (*I*^2^ = 87%, *p* = 0.0004), hence a random effects model was used. The meta-analysis showed that the Distal radius BMD at the last follow-up in the experimental group was significantly higher than in the control group. The difference was statistically significant (SMD = 1.27; 95% CI: 0.57,1.98; *p* = 0.0004; Figure 7).

##### 3.3.2.4 Metacarpal BMD

**Figure 8:**
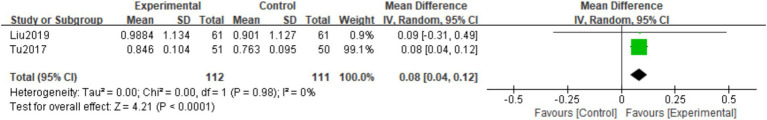
Forest plot of the Metacarpal BMD.

A total of 2 studies (19–25) observed Metacarpal BMD at the final follow-up. The homogeneity among the studies was good (*I*^2^ = 0%, *p* = 0.98), so a fixed effects model was used. Meta-analysis showed that the Metacarpal BMD at the final follow-up in the experimental group was significantly higher than in the control group. The difference was statistically significant (MD = 0.04; 95% CI: 0.04,0.12; *p* < 0.0001; Figure 8).

#### 3.3.3 Serum biochemical indexes

##### 3.3.3.1 Blood calcium

**Figure 9:**
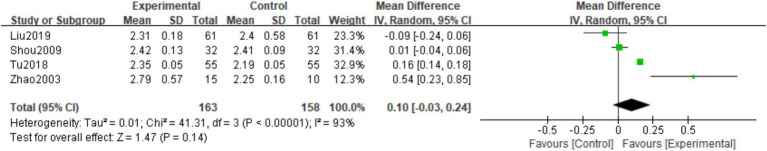
Forest plot of the Ca^2+^.

A total of four studies (16, 20, 21, 25) observed Ca^2+^ levels at the last follow-up. There was heterogeneity among the studies (*I*^2^ = 93%, *p* < 0.00001), so a random effects model was used. The meta-analysis showed that the Ca^2+^ levels at the last follow-up in the experimental group were higher than those in the control group. However, the difference was not statistically significant (MD = 0.10; 95% CI: −0.03,0.24; *p* = 0.14; Figure 9).

##### 3.3.3.2 Blood phosphorus

**Figure 10:**
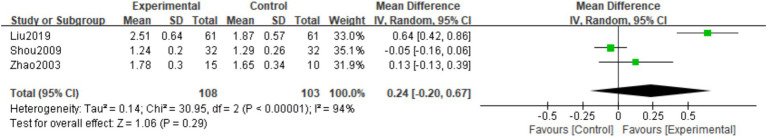
Forest plot of the P.

A total of three studies (16, 20, 25) observed P at the last follow-up. There was heterogeneity among the studies (*I*^2^ = 94%, p < 0.00001), so a random effects model was used. The meta-analysis showed that the P level at the last follow-up in the experimental group was higher than in the control group. However, the difference was not statistically significant (MD = 0.24; 95% CI: −0.20,0.67; *p* = 0.29; Figure 10).

##### 3.3.3.3 Serum alkaline phosphatase

**Figure 11:**
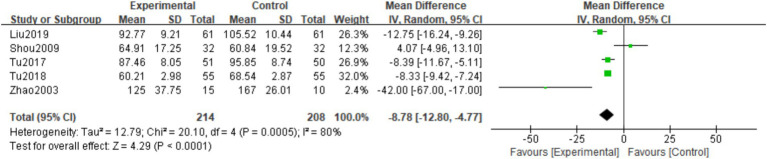
Forest plot of the ALP.

A total of five studies(16, 19–21, 25) observed ALP levels at the final follow-up. There was considerable heterogeneity among the studies (*I*^2^ = 80%, *p* = 0.0005), so a random effects model was used. The meta-analysis showed that the final follow-up ALP levels in the experimental group were lower than those in the control group. The difference was statistically significant (MD = -8.78; 95% CI: −12.80, −4.77; <0.0001; Figure 11).

##### 3.3.3.4 Bone alkaline phosphatase

**Figure 12:**
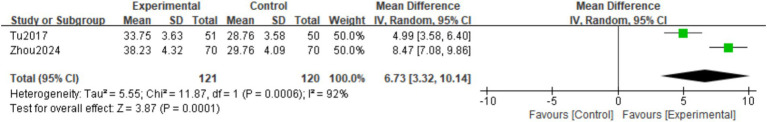
Forest plot of the BALP.

A total of two studies (19, 24) observed BALP at the final follow-up. There was significant heterogeneity among the studies (*I*^2^ = 92%, *p* = 0.0006), so a random effects model was used. The meta-analysis showed that the BALP levels at the final follow-up in the experimental group were higher than those in the control group. The difference was statistically significant (MD = 6.73; 95% CI: 3.32,10.14; *p* = 0.0001; Figure 12).

##### 3.3.3.5 Type I collagen cross-linked C-terminal peptide

**Figure 13:**
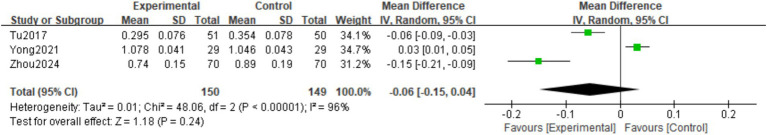
Forest plot of the CTX.

A total of three studies (18, 19, 24) observed the CTX at the last follow-up. There was heterogeneity among the studies (*I*^2^ = 96%, *p* < 0.00001), so a random effects model was used. The meta-analysis showed that the CTX levels at the last follow-up in the experimental group were lower than those in the control group. However, the difference was not statistically significant (MD = -0.06; 95%CI: −0.15,0.04; *p* = 0.24; Figure 13).

##### 3.3.3.6 Osteocalcin

**Figure 14:**
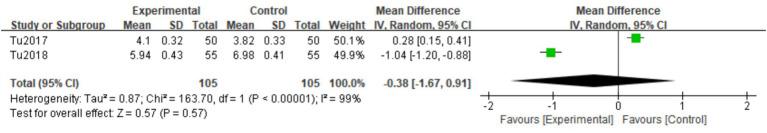
Forest plot of the BGP.

A total of two studies (19, 21) observed BGP at the final follow-up. There was heterogeneity among the studies (*I*^2^ = 99%, *p* < 0.00001), so a random effects model was used. The meta-analysis showed that the BGP level at the final follow-up in the experimental group was lower than in the control group. However, the difference was not statistically significant (MD = -0.38; 95% CI: −1.67,0.91; *p* = 0.57; Figure 14).

#### 3.3.4 Time to relieve lower back pain

**Figure 15:**
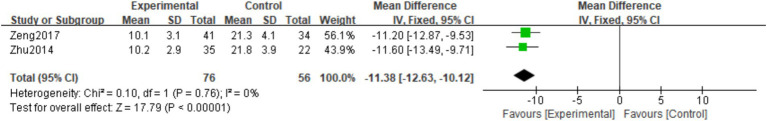
Forest plot of the time to relieve lower back pain.

A total of three studies (20, 22, 23) observed the time it took for patients to experience relief from lower back pain after treatment. Zhao et al. (20) only mentioned that the average time for the Epimedium group to see relief from lower back pain was 12 weeks, while the control group’s average time was 15 weeks. However, due to insufficient data, a meta-analysis could not be conducted. A meta-analysis was performed on the remaining two studies (22, 23). The homogeneity among the studies was good (*I*^2^ = 0%, *p* = 0.76), so a fixed effects model was used. The meta-analysis showed that the experimental group improved symptoms of lower back pain more quickly than the control group. The difference was statistically significant (MD = -11.38; 95% CI: −12.63, −10.12; *p* < 0.00001; Figure 15).

#### 3.3.5 Adverse events

Only two studies (18, 24) mentioned adverse reactions that occurred during the study. Zhou et al. (24) found that gastrointestinal reactions and skin allergic reactions were the main adverse reactions in both groups of patients during treatment. Among them, 4 out of 70 patients in the experimental group experienced stomach discomfort and bloating, and 4 had rashes and itching, with an adverse reaction rate of 5.71%; in the control group, 3 out of 70 patients experienced stomach discomfort and bloating, and 1 had a rash or itching, with an adverse reaction rate of 11.43%. However, there was no statistically significant difference in the incidence of adverse reactions between the two groups. Yong et al. (18) measured some liver and kidney function indicators and hematological markers over an 8-week observation period, finding levels exceeding normal ranges, such as one case of AST elevation (increased by 6%) observed in the third week; four participants showed increased ALP levels (over 30% above the normal upper limit) after taking EP, but all these changes subsequently returned to normal, without any substantial adverse clinical outcomes such as diarrhea, nausea, or vomiting. This suggests that the intake of total flavonoids from Epimedium not only does not cause adverse reactions but also does not increase the burden on liver and kidney functions. However, due to the limited reporting of adverse reaction events, more large-scale observations are still needed.

### 3.4 Sensibility analysis and publication bias

**Figure 16:**
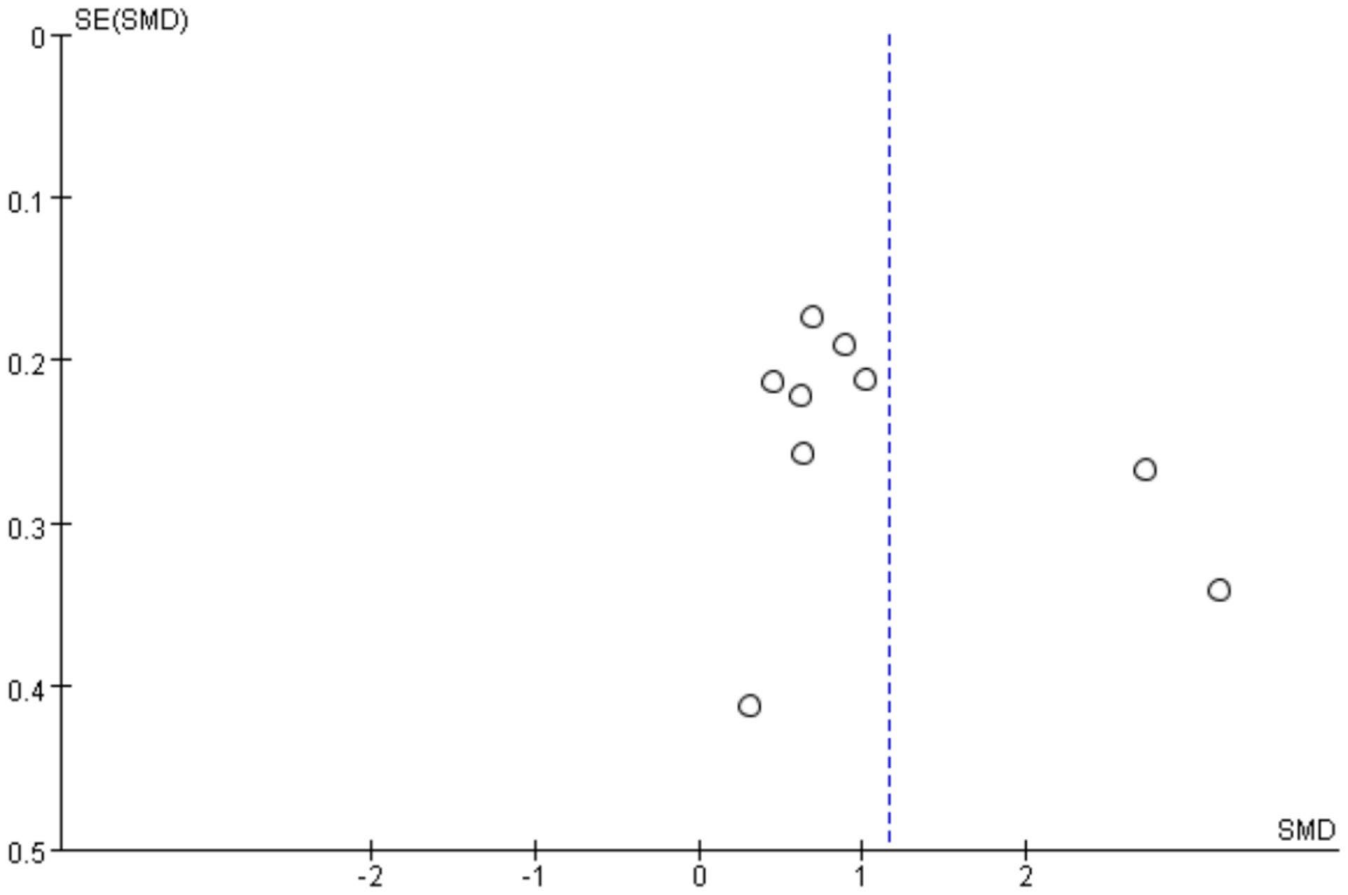
Bias risk of lumbar BMD.

**Figure 17:**
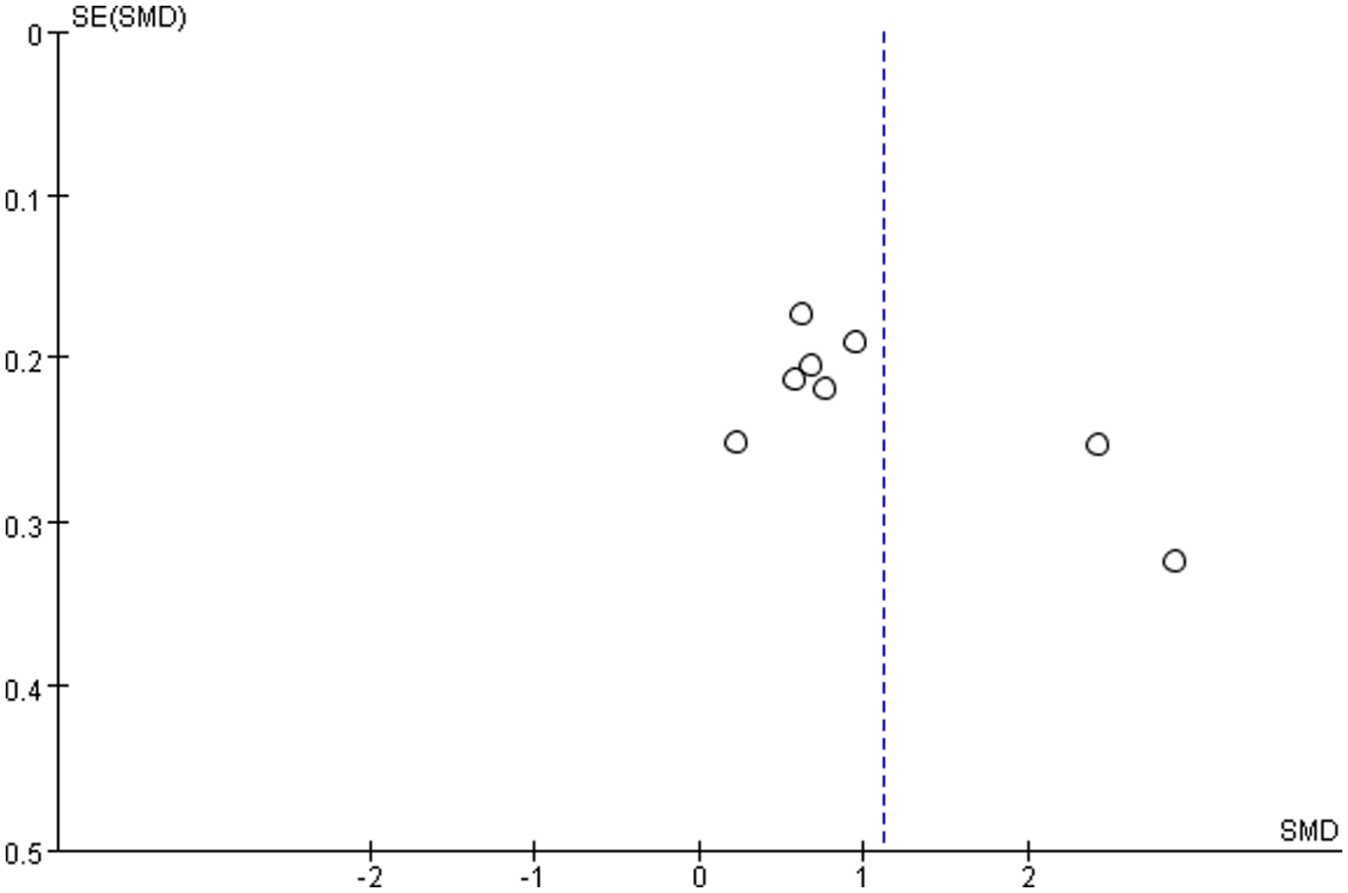
Bias risk of femoral neck BMD.

For the results with high heterogeneity, individual studies were excluded for sensitivity analysis to evaluate the impact of individual studies on the overall results. The results showed that omitting any one study from each comparison did not affect the overall effect, which verified the stability of this study. The funnel plot was used to test the publication bias of lumbar bone density and femoral neck bone density. Figures 16, 17 showed that the left–right distribution of each study in the figure was asymmetric, indicating that there may be potential publication bias in this study.

### 3.5 GRADE evidence evaluation results

The outcome indicators included in the design of this study, which consist of more than three studies, were evaluated for GRADE evidence levels. Among them, overall efficacy, lumbar bone density, and femoral neck bone density are considered moderate-quality outcome indicators, while Ca^2+^ and ALP are low-quality outcome indicators. For specific details, see Table 2. The rating results suggest that the quality of the relevant indicators involved in this study needs further improvement.

**Table 2:** GRADE evidence level evaluation table.

Outcome	Publication bias	Limitations	Indirectness	Inexactness	Inconsistency	Sample size	Effect size 95%CI	Quality of evidence
Total effective rate	Insignificant	Significant	Insignificant	Insignificant	Insignificant	457	OR = 3.80; 95%CI:2.27, 6.37; *p* = 0.0001	Medium quality
Lumbar BMD	Insignificant	Significant	Insignificant	Insignificant	Insignificant	817	SMD = 1.15; 95%CI:0.61, 1.70; *p*<0.0001	Medium quality
Femoral neck BMD	Insignificant	Significant	Insignificant	Insignificant	Insignificant	800	SMD = 1.11; 95%CI:0.58, 1.65; *p*<0.0001	Medium quality
Ca^2+^	Insignificant	Significant	Insignificant	Insignificant	Significant	321	MD = 0.10; 95%CI:-0.03, 0.24; p = 0.14	Inferior quality
ALP	Insignificant	Significant	Insignificant	Insignificant	Significant	312	MD = -8.78; 95%CI:-12.80, -4.77; *p*<0.0001	Inferior quality

## 4 Discussion

POP is a systemic bone disease characterized by reduced bone mass and degeneration of bone microstructure, primarily divided into postmenopausal OP and senile OP. Its pathogenesis involves aging, hormonal changes, and bone metabolic imbalance, particularly the decline in estrogen levels leading to increased osteoclast activity, where bone resorption exceeds bone formation, causing progressive bone density loss and an increased risk of fractures. Common symptoms include lower back pain, decreased height, and fragility fractures, significantly impacting quality of life. Epidemiological data show that postmenopausal women and the elderly are high-risk groups for POP, with a prevalence rate exceeding 30% in women, while although the incidence in men is lower, they have a higher risk of death after hip fracture. Globally, osteoporotic fractures result in nearly 10 million cases annually, not only degrading patients’ quality of life but also imposing a heavy economic burden on healthcare.

Modern medicine diagnoses and treats OP through bone density testing combined with clinical symptoms, focusing on calcium supplements, vitamin D, and the use of bisphosphonates to inhibit bone resorption or parathyroid hormone analogs to promote bone formation. Although current conventional treatments for POP can partially alleviate symptoms, long-term use has certain limitations. Long-term use of bisphosphonates may lead to atypical fractures or jaw necrosis, hormone replacement therapy carries risks of breast cancer and cardiovascular events, and the high cost of biologics limits their widespread use. These treatment methods often target a single point, making it difficult to comprehensively regulate the bone metabolic network, and long-term safety issues need to be addressed. In this context, Epimedium, a traditional Chinese herb for kidney tonification, demonstrates unique comprehensive advantages. Rich in flavonoids such as Epimedium glycosides, studies have found that it effectively enhances bone formation and reduces bone loss, regulating the bidirectional balance of bone metabolism to exert therapeutic effects. For postmenopausal OP, the plant estrogen-like action of Epimedium can partially compensate for estrogen deficiency, alleviating hyperostosis without the potential side effects of hormone replacement therapy. Its multi-target regulatory properties not only manifest in direct action on bone cells but also improve the microenvironment of bones through anti-inflammatory, antioxidant, antitumor, and bone metabolic regulatory pharmacological actions (27), synergistically enhancing the efficacy of traditional drugs. Clinical studies have shown that Epimedium extract or compound preparations can increase bone density in patients, reduce the incidence of vertebral fractures, and offer overall regulatory advantages in improving symptoms associated with kidney yang deficiency, such as soreness and weakness in the waist and knees (28). This demonstrates the organic combination of TCM’s theory of “tonifying the kidneys and strengthening bones” with modern pharmacological mechanisms, providing a safer and more comprehensive treatment option for the prevention and control of POP.

This study conducted a systematic review and meta-analysis to deeply explore the efficacy and safety of Epimedium in the treatment of POP. It strictly followed international norms such as PRISMA and comprehensively searched authoritative databases in both Chinese and English. Eventually, 10 RCTs that met the criteria were screened out, involving a total of 890 patients with POP. Comprehensive analysis shows that Epimedium preparations have significant advantages in improving the core clinical indicators of patients with POP. The core results of the meta-analysis showed that the total effective rate of the Epimedium group was significantly better than that of the control group (OR = 3.80; 95%CI: 2.27, 6.37), which means that patients treated with Epimedium were nearly four times more likely to achieve clinical efficacy than the control group, and this association was highly statistically significant. It is particularly worth noting that Epimedium performed outstandingly in rapidly alleviating the most common and life-affecting low back pain symptoms in patients with POP, with an average pain score significantly reduced by approximately 11.38 units compared to the control group (MD = -11.38; 95%CI: −12.63, −10.12). This reduction is not only statistically significant but also indicates clinically valuable pain relief, which is conducive to the early recovery of patients’ activity ability and quality of life. In clinical research, we also found that the combined application of Epimedium with bisphosphonates or calcium supplements demonstrated a good synergistic effect, suggesting that Epimedium can be used as an adjunctive treatment to enhance the efficacy of existing regimens. In addition, some of the included studies also suggest that Epimedium shows positive trends in increasing bone density and improving biochemical markers of bone metabolism.

The improvement in BMD was particularly notable. Meta-analysis results showed that at the final follow-up, the Lycium group had significantly higher lumbar bone mineral density (SMD = 1.15), femoral neck bone mineral density (SMD = 1.11), distal radius bone mineral density (SMD = 1.27), and metaphyseal joint bone mineral density (MD = 0.04) compared to the control group. Bone mineral density is a core indicator reflecting the degree of OP and fracture risk. These findings suggest that Lycium may improve bone metabolism through multiple targets. A SMD greater than 1.0 indicates a large effect size, especially the significant improvements in the lumbar spine and distal radius, which may be related to its rich content of lycoside and other flavonoids. These components have been confirmed by *in vitro* studies to promote osteoblastic differentiation and inhibit osteoclast activity through multiple targets and pathways. Notably, although the absolute value of the MD for the metaphyseal joint is small, its statistical significance (*p* < 0.05) remains clinically valuable, as a decrease of one standard deviation in BMD can increase the risk of fractures by 2.6 ~ 5.8 times (29). Notably, none of the studies included in our analysis reported fracture incidence. It is crucial to recognize that while BMD improvement serves as a significant intermediate indicator, it does not constitute a clinical primary endpoint. Although elevated BMD correlates with reduced fracture risk, this relationship is not linearly proportional. Therefore, our findings cannot directly confirm that Epimedium rhizome treatment effectively lowers fracture risks in patients with postoperative complications. The critical next step involves designing long-term, large-scale RCTs with fracture as the primary endpoint to provide definitive evidence of Epimedium’s clinical benefits. Additionally, there is high heterogeneity among studies (*I*^2^ = 87–92%), possibly due to differences in intervention methods (such as single extract versus compound formulations), treatment duration (3–24 months), and population characteristics (such as age and gender ratio). It is noteworthy that while this study identified potential sources of high heterogeneity such as intervention methods, treatment duration, and population characteristics, the limited number of included studies (*n* = 10) and the absence of detailed subgroup data in original literature (e.g., gender, age stratification, baseline bone density values) prevented us from conducting quantitative subgroup analysis to verify their impact on therapeutic efficacy. Such differences may significantly affect the clinical applicability of Epimedium preparations. Future research urgently requires prospective stratified designs and standardized reporting of subgroup data to clarify the clinical efficacy and applicability of Epimedium.

The meta-analysis results of bone metabolism markers in this study reveal the bidirectional mechanism by which Epimedium regulates bone metabolism, and its profound significance deserves further interpretation. The significant decrease in serum ALP (MD = -9.30, *p* = 0.01) was not only statistically significant, but also the extent of the reduction was closer to the clinically significant threshold. As an indirect marker of the bone resorption process, the decline of ALP strongly suggests that Epimedium can effectively inhibit the activation of osteoclasts and bone matrix dissolution. This effect may be achieved by regulating the imbalance of the nuclear factor κB receptor activator ligand/osteoprotegatin (RANKL/OPG) signaling axis - basic research shows that icaliin can down-regulate the expression of RANKL At the same time, it promotes the secretion of OPG, thereby blocking the osteoclast differentiation pathway (30). More specifically, there was a significant increase in BALP (MD = 6.73, *p* = 0.0001), which directly reflects the activity of osteoblasts and the activity level of the bone mineralization frontier. Studies have shown that icariin promotes the differentiation of BMSCs into osteoblasts by activating the Wnt/*β*-catenin signaling pathway, while inhibiting their differentiation into adipocytes (31). In addition, icariin can also promote osteogenic differentiation by activating the BMP/Smads/Runx2/Osx signaling pathway and up-regulating the expression levels of Runx2 and COL-1 (32, 33). This bidirectional regulatory model of “inhibiting bone resorption - promoting bone formation” breaks through the single-phase effect limitation of traditional anti-bone resorption drugs and reflects the multi-target regulation characteristics of TCM. While basic research indicates that Ipratoposide regulates bone metabolism through multiple pathways, none of the included clinical studies measured active compound concentrations in patients’ serum or tissues. Given its low oral bioavailability and complex metabolic processes (34, 35), attributing clinical efficacy to specific molecular pathways remains a theoretical hypothesis. Future pharmacokinetic-therapeutic studies are needed to verify the correlation between target exposure levels and therapeutic outcomes.

It is worth noting that the levels of blood calcium, blood phosphorus, CTX and BGP did not reach statistical significance and need to be comprehensively analyzed in combination with methodological and biological essence. Take type I collagen cross-linked CTX as an example. As the most sensitive marker of bone resorption, its negative results are mainly restricted by three aspects: Firstly, only three studies have reported this indicator, the cumulative sample size is less than 200 cases, and the statistical testing power is significantly insufficient (Post-hoc power<0.5). Secondly, CTX detection is significantly influenced by circadian rhythm and eating status, but most of the included studies did not standardize the blood collection process (such as not stipulating fasting in the morning) (36). Thirdly, Epimedium may preferentially act on the early bone resorption stage (such as inhibiting the fusion of osteoclast precursor cells), while its effect on the CTX secreted by mature osteoclasts lags behind (37). Furthermore, research indicates that BGP is a specific indicator reflecting the rate of bone formation. Its serum concentration is positively correlated with BGP in bone tissue and is not affected by the status of vitamin K. As a late marker of bone matrix mineralization, BGP is secreted in large numbers only in the later stage of osteoblast differentiation. However, most studies have an intervention period of ≤6 months, which is precisely during the “rapid response period” of bone metabolic remodeling - at this stage, osteoblasts are still in the proliferation/differentiation stage (reflected as elevated BALP). It has not yet entered the mineralization period of large-scale synthesis of bone matrix (the peak release of BGP often occurs 9 to 12 months after treatment) (38). Therefore, during the rapid response period of bone metabolism, the level of BGP may not have reached the detectable threshold yet, thereby leading to the occurrence of negative results. This phenomenon holds significant physiological importance in clinical and experimental research, suggesting that when evaluating bone metabolism, we should fully consider the secretion kinetics characteristics of BGP to avoid drawing incorrect conclusions due to short-term detection. This suggests that future research needs to design longer observation Windows and adopt dynamic monitoring strategies (such as detecting marker profiles every 3 months) to capture the phased characteristics of bone metabolism.

The clinical application advantages of Epimedium are not only reflected in its therapeutic effect, but also in its prominent safety features. Meta-analysis showed that the included studies generally reported good short-term tolerance of Epimedium, no severe liver or kidney toxicity or complications common with traditional drugs were observed, and the incidence of common minor adverse reactions (such as gastrointestinal discomfort) was low, with no significant difference compared with the control group. Compared with traditional anti-OP drugs, the safety characteristics of Epimedium show multi-dimensional advantages: For instance, the incidence of flu-like symptoms such as joint pain, headache and arthralgia caused by bisphosphonates is as high as 54.30%, and it may also induce atypical femoral fractures and jawbone necrosis (8). Hormone replacement therapy (HRT) increases the risk of breast cancer and cardiovascular events, etc. (9), while the high cost of biological agents limits their wide application. Epimedium has not yet been clearly associated with these serious complications. Considering the accessibility of its raw materials and the cost of its formulation, it has significant cost-effectiveness and patient compliance advantages in long-term disease management. It is worth noting that Zhang et al. (17) demonstrated that the estrogen-like effect of Epimedium is achieved through the selective activation of ERα receptors, which not only simulates the protective effect of estrogen on bones but also avoids the potential carcinogenic risks of hormone replacement therapy to the breast and endometrium. It is particularly suitable for postmenopausal OP, a pathological state driven by estrogen deficiency. The findings of Yong et al. (18) are even more intriguing: although transient fluctuations in liver enzymes (ALT/AST) or creatinine were observed, these changes remained within the “laboratory anomalies” category (such as ALT<80 U/L), did not progress to the pathological damage threshold, and could spontaneously return to normal within 2 to 4 weeks after drug withdrawal. This indicates that the intake of total flavonoids from Epimedium does not cause adverse reactions and does not increase the burden on liver and kidney functions. However, recent toxicological studies highlight potential hepatotoxicity concerns that warrant attention. Research indicates that the Chinese herbal medicine Epimedium may exert indirect toxicity. Its metabolized components or drug interactions can stimulate immune responses, exacerbating liver inflammation and causing hepatocyte-dominant liver damage (39). However, this effect shows a dose-dependent relationship. Whether conventional clinical doses would lead to liver injury depends on the continuous advancement of understanding and research into the pharmacological properties of TCM. In addition, the safety of synergistic interactions between Epimedium and other drugs also needs to be evaluated. A comprehensive lipidomics and transcriptomics study revealed that the active ingredients of Epimedium (lcariin) and those of *Psoralea corylifolia* (Bavachin) synergistically induce specific liver injury (40). Crucially, clinical studies in this meta-analysis reported no severe hepatotoxicity, with transient enzyme fluctuations normalizing post-treatment. The long-term safety of Epimedium, especially its potential effects on liver and kidney functions and the endocrine system, as well as its impact on the most serious consequence of OP - the incidence of fractures, still needs to be verified through longer-term, large-sample prospective studies.

The limitations of this study need to be fully considered at the level of evidence-based practice. Firstly, the 10 RCTs included, although they constitute the current evidence base, have obvious shortcomings in their methodological quality: Most studies did not describe in detail the specific methods for generating random sequences (such as random number tables or computer programs), more than half of the trials did not implement allocation hiding (such as not using sealed envelopes or central randomization systems), and only a few studies adopted double-blind designs (especially lacking blinds for outcome evaluators). These defects may lead to selection bias and implementation bias, affecting the reliability of the results. At the same time, this study may have publication bias. Usually, positive results with small sample size are easier to be published. Although the sensitivity analysis shows that the results are robust, the high risk of bias may still overstate the effect size, so the results should be interpreted carefully. Secondly, the clinical heterogeneity among the studies deserves attention - there are differences in extracts, compound decoctions and different dosage forms of Epimedium preparations, with varying dosages and treatment courses ranging from 2 months to 1 year. Meanwhile, the intervention measures in the control group were not uniform, ranging from basic calcium supplementation to active anti-bone resorption drugs. The diversity of this “intervention-control” combination leads to ambiguity in efficacy attribution and makes it difficult to determine the monotherapy contribution of Epimedium. More crucially, the research on the mechanism is weak: Currently, the synergistic effect of Epimedium with other drugs (such as bisphosphonates or teriparatide) is still at the clinical observation stage, lacking quantitative pharmacodynamic studies on key pathways, resulting in the optimal compatibility plan and dose window not being clearly defined. The most prominent limitation lies in the short-term nature of the endpoint indicators: the longest follow-up for the included studies was only 52 weeks, while the core goal of OP treatment - reducing the risk of fractures - usually takes 3 to 5 years of observation to show. Meanwhile, the lack of long-term monitoring data (≥2 years) on liver and kidney metabolic indicators, coagulation function and endocrine hormones makes it difficult to comprehensively assess the safety of the drug. Although these limitations do not deny the reference value of the existing conclusions, they suggest that in clinical application, decisions should be made with caution based on the individual characteristics of patients, and it is expected that more solid evidence will be provided through high-quality long-term studies in the future.

Future research directions should focus on the following points: First, design large-scale, long-term RCTs to clarify the impact of Epimedium on the incidence of osteoporotic fractures. Second, explore the combined application effect and mechanism of Epimedium with other anti-OP drugs such as teriparatide, denosumab and Chinese medicine. Third, combine metabolomics, genomics and other multi-omics technologies to screen sensitive populations for Epimedium treatment and promote individualized drug use. Fourth, establish quality control standards for Epimedium preparations to ensure the stability and consistency of active ingredients. Additionally, it is necessary to strengthen integrated traditional Chinese and Western medicine research, combining the traditional theory that “kidney governs bones” with modern bone metabolism regulatory networks to deepen understanding of Epimedium’s “multi-component, multi-target, multi-pathway” action mode.

## 5 Conclusion

This meta-analysis confirms that Epimedium as an adjunct to conventional therapy significantly improves BMD, alleviates pain, and regulates bone metabolism markers in POP patients, with favorable safety profiles. Its mechanism of action may involve inhibiting ALP and promoting BALP to achieve bidirectional regulation of bone resorption and formation. However, given the small sample size and generally low quality of included literature, some studies show significant heterogeneity, which requires cautious interpretation. Future large-scale, long-term follow-up clinical trials are needed to clarify the reduced fracture risk associated with Epimedium macrocarpon, explore the mechanism of its action as a single drug or synergistic effect with other preparations, and establish standardized quality control for preparations, providing a basis for the use of Epimedium in treating POP.

## Data Availability

The original contributions presented in the study are included in the article/supplementary material, further inquiries can be directed to the corresponding author/s.

## References

[1] Zhang ZL. Osteoporosis and bone mineral diseases branch of the Chinese medical association. Diagnosis and treatment guidelines for primary osteoporosis (2022). Chin Gen Pract. (2023) 26:1671–91. doi: 10.3969/j.issn.1674-2591.2022.06.001

[2] Botege XA. Epidemiological research and prevention and treatment strategies of primary osteoporosis. Health Care Today. (2024) 24:1273–5. doi: 10.3969/j.issn.1671-0223(s).2024.16.024

[3] Hernlund E Svedbom A Ivergård M Compston J Cooper C Stenmark J . Osteoporosis in the European Union: medical management, epidemiology and economic burden. A report prepared in collaboration with the international osteoporosis foundation (IOF) and the European Federation of Pharmaceutical Industry Associations (EFPIA). Arch Osteoporos. (2013) 8:136. doi: 10.1007/s11657-013-0136-1 24113837 PMC3880487

[4] Zhou Y Zhang DY Wu LF Wang G Mu J Cui C . Epidemiological investigation of osteoporosis in Beijing area in the past 10 years: analysis of bone mineral density examination results of 30,599 Han people in a single center by dual-energy X-ray absorptiometry. J South Med Univ. (2025) 45:443–52. doi: 10.12122/j.issn.1673-4254.2025.03.01 PMC11955889 40159958

[5] Chen Z Wen Y Qiu M Fang L Jin O Gu J. The pattern and trends of disease burden due to low bone mineral density from 1990 to 2019 in China: findings from the global burden of disease study 2019. Arch Osteoporos. (2022) 17:79. doi: 10.1007/s11657-022-01079-9 35247103

[6] Srivastava M Deal C. Osteoporosis in elderly: prevention and treatment. Clin Geriatr Med. (2002) 18:529–55. doi: 10.1016/s0749-0690(02)00022-8 12424871

[7] Wang QQ. Progress in drug treatment of primary osteoporosis. Zhejiang Med J. (2021) 24:673–81. doi: 10.12056/j.issn.1006-2785.2024.46.7.2024-407

[8] Liang YH. Research and progress of bisphosphonate drug-related osteonecrosia of the jawbone. Med Innov China. (2024) 21:180–4. doi: 10.3969/j.issn.1674-4985.2024.11.039

[9] Kanis JA Cooper C Rizzoli R, Reginster JYScientific advisory board of the European society for clinical and economic aspects of osteoporosis (ESCEO) and the committees of scientific advisors and national societies of the international osteoporosis foundation (IOF). European guidance for the diagnosis and management of osteoporosis in postmenopausal women. Osteoporos Int. (2019) 30:3–44. doi: 10.1007/s00198-018-4704-5 PMC7026233 30324412

[10] Fan KJ Niu AW Wu HH. Epimedium glycosides promote the proliferation and differentiation of MC3T3-E1 cells through the PI3K/AKT signaling pathway. Cent S Univ Pharm. (2025) 23:417–22. doi: 10.7539/j.issn.1672-2981.2025.02.019

[11] Zhang H Zheng YL Ding H. Research progress on the related pathways of icariin in the treatment of osteoporosis. J Tianjin Univ Tradit Chin Med. (2022) 41:531–8. doi: 10.11656/j.issn.1673-9043.2022.04.22

[12] Zu Y Mu Y Li Q Zhang ST Yan HJ. Icariin alleviates osteoarthritis by inhibiting NLRP3-mediated pyroptosis. J Orthop Surg Res. (2019) 14:307. doi: 10.1186/s13018-019-1307-6 31511005 PMC6737611

[13] Gan JW Xie BP Liao XF. Study on the inhibition of osteoclast differentiation and bone resorption by icariin through estrogen receptor. Jiangxi Med J. (2022) 57:1378–80. doi: 10.3969/j.issn.1006-2238.2022.10.012

[14] He LJ Huang QW Ma HH Zhang L Zhang Y Wang YZ. The mechanism of Epimedium glycosides protecting osteoporosis in ovariectomized rats through autophagy and apoptosis pathways. New Tradit Chin Med Drugs Clin Pharmacol. (2023) 34:149–55. doi: 10.19378/j.issn.1003-9783.2023.02.002

[15] Moher D Liberati A Tetzlaff J Altman DGThe PRISMA Group. Preferred reporting items for systematic reviews and meta-analyses: the PRISMA statement. PLoS Med. (2009) 6:e1000097. doi: 10.1371/journal.pmed.1000097 19621072 PMC2707599

[16] Shou ZX Shen L Yang YP Xie J Zhou PQ Gao L. Effects of epimedium total flavonoids on bone mineral density and bone metabolism-related indices in primary osteoporosis. J Clin Rehabil Tissue Eng Res. (2009) 13:2191–5.

[17] Zhang G Qin L Shi Y. Epimedium-derived phytoestrogen flavonoids exert beneficial effect on preventing bone loss in late postmenopausal women: a 24-month randomized, double-blind and placebo-controlled trial. J Bone Miner Res. (2007) 22:1072–9. doi: 10.1359/jbmr.070405 17419678

[18] Yong EL Cheong WF Huang Z Thu WPP Cazenave-Gassiot A Seng KY . Randomized, double-blind, placebo-controlled trial to examine the safety, pharmacokinetics and effects of Epimedium prenylflavonoids, on bone specific alkaline phosphatase and the osteoclast adaptor protein TRAF6 in post-menopausal women. Phytomedicine. (2021) 91:153680. doi: 10.1016/j.phymed.2021.153680 34352588

[19] Tu Y Xiong LN Liu XJ Shen Y Qin YQ. Clinical study on the treatment of primary osteoporosis with compound Epimedium oral liquid. J Chin Med. (2017) 32:1981–4. doi: 10.16368/j.issn.1674-8999.2017.10.520

[20] Zhao LN. Clinical effect evaluation of Epimedium macrocarpon in the prevention and treatment of osteoporosis. J Integr Tradit Chin West Med. (2003) 9:922–3. doi: 10.3969/j.issn.1008-8849.2003.09.015

[21] Tu Y Xiong LN Liu XJ Lei C Li NX Zhang RY. Clinical effect observation of epimedium combined with Danshen Kang bone capsules in the treatment of senile osteoporosis. Anhui Yi Ke Da Xue Xue Bao. (2018) 22:1814–7. doi: 10.3969/j.issn.1009-6469.2018.09.047

[22] Zhu GC Liu MY Cai Z Sun LF Song XH. Clinical observation of treating senile osteoporosis with epimedium and calcium d. Hebei Yi Xue. (2014) 36:183–4. doi: 10.3969/j.issn.1002-7386.2014.02.007

[23] Zeng X Tang Q Wang LJ Tan T Shi L Niu XD. Clinical efficacy of Epimedium in the treatment of osteoporosis. J Ration Clin Drug Use. (2017) 10:101–2. doi: 10.15887/j.cnki.13-1389/r.2017.09.049

[24] Zhou YJ Wang WW Lian KQ Wang B Lin D. Clinical study on the combined use of Epimedium total flavonoid capsules and alendronate for treating primary osteoporosis (kidney yang deficiency syndrome). Mod Drugs Clin. (2024) 39:2902–7. doi: 10.7501/j.issn.1674-5515.2024.11.027

[25] Liu HY. Clinical observation of Chinese medicine Epimedium to treat osteoporosis. Inner Mongolia Tradit Chin Med. (2019) 38:16–7. doi: 10.16040/j.cnki.cn15-1101.2019.01.012

[26] Zhou XJ Yao XM Zhou YY. Advances in the study of the pharmacological effects of Epimedium. J Tradit Chin Med. (2022) 50:112–5. doi: 10.19664/j.cnki.1002-2392.220262

[27] Lv NN Zhang H Feng XX Liu MM. Epimedium glycoside intervention research progress of osteoporosis. J Jiangsu Univ. (2022) 32:22–5. doi: 10.13312/j.issn.1671-7783.y210086

[28] Wan YN Li S Jiang YX Chen WH Jia HM Che RW. Research progress on the treatment of glucocorticoid induced osteoporosis with Epimedium brevicornum and its compound preparations. Chin J Osteoporos. (2019) 25:713–6. doi: 10.3969/j.issn.1006-7108.2019.05.027

[29] Li CZ Pang YX Yu L Tang X. The value of bone mineral density in risk assessment of hip osteoporotic fracture. Chin J Osteoporos. (2020) 26:1023–7. doi: 10.3969/j.issn.1006-7108.2020.07.017

[30] Li SB Xia T Zhang XY Wang WW Zhou Y Lai Y. The active monomer components of Epimedium regulate the osteoporosis-related signaling pathways and affect the homeostasis of bone resorption and bone formation. Zhongguo Zuzhi Gongcheng Yanjiu. (2022) 26:1772–9.

[31] Li JJ Xia T Liu JM Chen F Chen HT Zhuo YH . Molecular mechanism of icariin regulating osteogenic signal-related pathways in the treatment of steroid-induced avascular necrosis of the femoral head. Zhongguo zu zhi gong cheng yan jiu. (2022) 26:780–5.

[32] Hou XF Gao YH Bai X Chen KM. Research progress on the mechanism of icariin promoting fracture healing. Biomed Transl Sci. (2021) 2:89–94. doi: 10.12287/j.issn.2096-8965.20210113

[33] Wang DX Xu ZW Pei GX. Osteogenesis of bone marrow mesenchymal stem cells on the icariin/hydroxyapatite/polylactic acid-glycolic acid copolymer scaffold. Zhongguo Zu Zhi Gong Cheng Yan Jiu. (2020) 24:3974–80. doi: 10.3969/j.issn.2095-4344.2083

[34] Lin TT Li XC Qiu HW Liu ZQ Zhu LJ. Safety evaluation of hypoglycemic components in Epimedium breviscapus and pharmacokinetic study of its five main components. Tradit Chin Med New Drug Clin Pharmacol. (2024) 35:402–10. doi: 10.19378/j.issn.1003-9783.2024.03.012

[35] OuYang HZ He J. Progress in chemical composition analysis and pharmacokinetics of Epimedium brevicornum. J Tianjin Univ Tradit Chin Med. (2019) 38:219–27. doi: 10.11656/j.issn.1673-9043.2019.03.04

[36] Dal Prá KJ Lemos CA Okamoto R Soubhia AM Pellizzer EP. Efficacy of the c-terminal telopeptide test in predicting the development of bisphosphonate-related osteonecrosis of the jaw: a systematic review. Int J Oral Maxillofac Surg. (2017) 46:151–6. doi: 10.1016/j.ijom.2016.10.009 27876532

[37] Zou JY Peng Y Wang Y Zou J Xu S Shi C . Icariin ameliorates osteoporosis by activating autophagy in ovariectomized rats. Adv Clin Exp Med. (2024) 33:941–52. doi: 10.17219/acem/174078 38235994

[38] Zhang MM Ma QQ Mao WX. Expert consensus on the clinical application of biochemical indicators of bone metabolism (revised edition 2023). Chin J Osteoporos. (2023) 1:1–14. doi: 10.3969/j.issn.1006-7108.2020.06.001

[39] Wang ZL. Study on the immunodiversity susceptibility components and mechanisms of Epimedium brevicornum-induced liver injury based on NLRP3 inflammasome. Chengdu University of Traditional Chinese Medicine. (2021). doi: 10.26988/d.cnki.gcdzu.2021.000375

[40] Li YY Cao B Lin MM Xu J Qi S Wang J . An integrative lipidomics and transcriptomics study revealing Bavachin and icariin synergistically induce idiosyncratic liver injury. Immunopharmacol Immunotoxicol. (2024) 46:924–34. doi: 10.1080/08923973.2024.2424293 39505304

